# The lasting burden of gender discrimination in medicine: lifelong multisystem consequences of Rathke’s pouch resection in Williams syndrome

**DOI:** 10.1097/JS9.0000000000004759

**Published:** 2026-02-04

**Authors:** Abubakar Nazir, Muhammad Saad, Imran Naqvi

**Affiliations:** a Department of Medicine, The Jewish Hospital-Mercy Health, Cincinnati, Ohio, USA; b Oli Health Magazine Organization, Kigali, Rwanda

*Dear Editor,*

The latter half of the 20th century witnessed significant advancements in surgical and medical interventions; however, it was also characterized by persistent gender-based disparities that profoundly impacted women with complex or rare conditions. Historical biases often led to delayed diagnoses, inappropriate interventions, and systemic neglect, particularly when patients presented with atypical or syndromic phenotypes^[1,2]^. Women’s symptomatology especially reproductive and pain-related complaints was frequently minimized or dismissed, and clinical decision-making was often guided by societal assumptions rather than evidence-based practice. Rare syndromes, such as Williams syndrome (WS), were frequently under-recognized, and interventions addressing acute pathology frequently failed to account for long-term functional and psychosocial consequences^[1,2]^. This study was conducted and reported in compliance with the TITAN 2025 Guidelines on transparent integration of AI in academic work in line with current publishing standards on responsible AI usage and reporting^[3]^.

This context is exemplified by a 62-year-old female with WS who underwent a Rathke’s pouch resection at age 16 in the 1960s. Prior to surgery, she had normal menarche and cyclic menstruation. Following the procedure, she developed permanent amenorrhea, resulting in lifelong infertility and profound psychosocial consequences, including social isolation and inability to marry. She also demonstrated developmental delays not observed among family members. Her phenotype was consistent with classical WS, including craniofacial dysmorphism (widely spaced teeth, long philtrum, flattened nasal bridge), microcephaly, hypotonia, and failure to thrive during infancy^[4]^. Despite these salient features, delayed recognition of her syndrome and prevailing gendered assumptions likely contributed to suboptimal management and lifelong sequelae. The long-term consequences of early pituitary surgery were most apparent in reproductive and endocrine function. Permanent amenorrhea and infertility represent irreversible outcomes of intervention during adolescence, a period of critical hormonal and reproductive maturation. These outcomes underscore historical neglect of female reproductive health and lack of longitudinal consideration in perioperative endocrine evaluation. In adulthood, the patient developed primary hypothyroidism, with suppressed TSH and low free T4, likely reflecting combined primary thyroid dysfunction and pituitary insufficiency, further emphasizing the multisystem consequences of early surgical intervention in WS^[4]^. This patient presented acutely with hypertensive emergency (BP 197/113 mmHg), elevated troponin (initial 150 ng/L, repeat 205 ng/L), and pulmonary edema. Chronic cardiovascular risk is compounded by longstanding hypertension and tobacco use. These findings exemplify how early neglect and under-recognition of syndromic cardiovascular risks can precipitate acute morbidity decades later.

Her neurological history includes left frontal cavernoma, prior subarachnoid hemorrhage (2016), and Rathke’s pouch resection. On presentation, she experienced severe, radiating headache with posterior neck involvement. MRI revealed Chiari I malformation with 13-mm tonsillar ectopia, a small C2-C3 syrinx, and a possible additional syrinx near the inferior cerebellar peduncle. Imaging also confirmed chronic left frontal subarachnoid hemorrhage, highlighting persistent CNS vulnerability and long-term neurological sequelae of early interventions without comprehensive syndromic evaluation.

She had cervical rigidity, accentuated lordotic alignment, and degenerative changes at multiple cervical levels, with pain exacerbated by movement and axial loading, consistent with hypotonia and chronic biomechanical stress inherent to WS^[4]^. This emphasizes the need for longitudinal musculoskeletal assessment in patients with rare syndromes and prior neurosurgical interventions.

The devastating consequences was that at 62 years of age, she remains single, homeless, and without employment throughout the life, her social world constricted by the lifelong shadows of chronic developmental delays and irreversible infertility. Each of these outcomes the loss of reproductive potential, the thwarted personal relationships, the absence of familial support represents not merely a statistic but a profound human cost, a life shaped by decisions made in an era when women’s voices and complex medical realities were routinely marginalized. Her story lays bare the interwoven medical, social, and psychological toll exacted by historical gender-based neglect, serving as a haunting testament to the lives imperiled when the healthcare system fails to recognize, validate, and protect women with rare syndromes. Her experience underscores the enduring consequences of historical inequities, including permanent reproductive loss, chronic multisystem morbidity, and psychosocial adversity. Reflecting on such cases provides critical insight for modern medicine, emphasizing the need for equitable, informed, and holistic approaches to care for women with complex or rare conditions and ensuring that historical biases do not perpetuate preventable morbidity in future generations^[2]^.

Women in the 20th century, particularly those with complex phenotypes, were disproportionately vulnerable to delayed diagnoses, inappropriate interventions, and systemic neglect^[1,2]^. Recognition of these historical injustices is essential to inform contemporary practice. Multidisciplinary care including endocrinology, cardiology, neurology, rehabilitation, and psychosocial support is critical for patients with rare syndromes. Awareness of historical gender bias reinforces the necessity of evidence-based, patient-centered care, longitudinal monitoring, and proactive management of reproductive, developmental, and multisystem health^[1]^.

## Data Availability

Not applicable.

## References

[1] Lassi ZS Wade JM Ameyaw EK. Stages and future of women’s health: a call for a life-course approach. Womens Health Lond Engl 2025;21:17455057251331721. 40258196 10.1177/17455057251331721 PMC12035259

[2] Khasriya R Horsley H. Why women are not treated equally in healthcare and what can be done. Nat Rev Urol 2025:1–3. doi: 10.1038/s41585-025-01063-1. 40562849 10.1038/s41585-025-01063-1

[3] Agha RA Mathew G Rashid R. Transparency in the reporting of artificial intelligence–the TITAN guideline. Prem J Sci 2025;10:100082.

[4] Kozel BA Barak B Kim CA. Williams syndrome. Nat Rev Dis Primers 2021;7:42. 34140529 10.1038/s41572-021-00276-z PMC9437774

